# Different Cellular Response of Human Mesothelial Cell MeT-5A to Short-Term and Long-Term Multiwalled Carbon Nanotubes Exposure

**DOI:** 10.1155/2017/2747215

**Published:** 2017-08-08

**Authors:** Li Ju, Wei Wu, Min Yu, Jianlin Lou, Hao Wu, Xianhong Yin, Zhenyu Jia, Yun Xiao, Lijin Zhu, Jun Yang

**Affiliations:** ^1^Zhejiang Academy of Medical Sciences, Hangzhou, China; ^2^Jiading District Center for Disease Control and Prevention, Shanghai, China; ^3^Hangzhou Normal University School of Public Health, Hangzhou, China

## Abstract

Despite being a commercially important product, multiwalled carbon nanotubes (MWCNTs) continue to raise concerns over human health due to their structural similarity to asbestos. Indeed, exposure to MWCNT has been shown to induce lung cancer and even mesothelioma, but contradictory results also exist. To clarify the potentially carcinogenic effects of rigid and rod-like MWCNT and to elucidate the underlying mechanisms, the effects of MWCNT on human mesothelial cell MeT-5A were examined throughout 3 months of continuous exposure, including cytotoxicity, genotoxicity, and cell motility. It was found that MWCNT did not affect MeT-5A cell proliferation at 10 *μ*g/cm^2^ within 72 h treatment, but under the same condition, MWCNT induced genotoxicity and perturbed cell motility. In addition, MeT-5A cells demonstrated different cellular responses to MWCNT after short-term and long-term exposure. Taken together, our results indicated a possible carcinogenic potential for MWCNT after long-term treatment, in which Annexin family proteins might be involved.

## 1. Introduction

Multiwalled carbon nanotubes (MWCNTs) have been subjected to widespread applications in many areas such as biomedical, engineering, and material science areas, owing to their extraordinary physical, chemical, and optical properties. However, due to their needle-like shape and high durability, concerns over human health have been raised that MWCNT may induce asbestos-like pathogenicity. Indeed, many publications have shown that MWCNT could induce mesothelioma in rodents and exhibit genotoxic effects in various cell systems. For example, MWCNT caused genotoxic injury in different cell lines, leading to the induction of high mobility group box-1 protein (HMGB1), reactive oxygen species (ROS), or hypoxanthine-guanine phosphoribosyl transferase (HPRT) mutations [1]. Dose-dependent increase of DNA strand breaks was also observed in lung and bronchoalveolar lavage (BAL) cells in mice exposed to aerosolized MWCNT [2]. Furthermore, fully characterized MWCNT were able to induce epithelial-mesenchymal transition (EMT) in human bronchial epithelial cells (BEAS-2B), and a neoplastic-like transformation was demonstrated by increased cell proliferation, anchorage-independent growth, invasion, and angiogenesis in human lung epithelial cells (SAECs) at occupationally relevant concentrations [3]. Moreover, it was reported that MWCNT could cause lung cancer and mesothelioma in rats in vivo [4, 5] and induce DNA damage in rat lung cells and DNA damage lasted even 90 days after exposure [6]. Recently, MWCNT-7, rigid and rod-like, is classified as a Group 2B carcinogen with sufficient evidence of carcinogenicity in animals and possible carcinogenicity to humans, by WHO/International Agency for Research on Cancer (WHO/IARC). But, data available to date could not determine the same classification for other types of carbon nanotubes (CNTs), and the potential molecular mechanism has yet to be fully elucidated.

Contradictory findings regarding the carcinogenicity of MWCNT have also been shown in some studies. For instance, no induction of DNA damage was observed in A549 and BEAS-2B cells after NM-401, NM-402, and NM403 MWCNT treatments by the comet assay [7, 8]. No systemic genotoxic effects were observed for MWCNT in leukocytes, bone marrow, or blood, as assessed by the *γ*-H2AX assay or micronucleated polychromatic erythrocytes (MNPCEs) [2, 9]. The reason of the inconsistent biological effects may be imputable to the varied types of MWCNTs, different experimental condition, or different end points.

As noted above, more thorough, in-depth examination is urgently needed to identify the carcinogenic ability and mechanisms for most types of CNTs, in an effort to reduce the uncertainty and prevent the potential damage in future. Therefore, in the present study, the human mesothelial cell MeT-5A was employed to detect the cellular response to MWCNT throughout 3 months of exposure, including cytotoxicity, genotoxicity, and cell motility. As reported here, MeT-5A cells demonstrated different cellular response to short-term and long-term exposure of MWCNT.

## 2. Materials and Methods

### 2.1. MWCNT Preparation

MWCNTs (Aldrich 659258) were obtained from Sigma-Aldrich (St Louis, Missouri). Their main characteristics have been previously described and summarized [10, 11]. Briefly, their dimensions (diameter × length) were 110 to 170 nm × 5 to 9 *μ*m, the purity was more than 90% with metal contaminants, mostly iron (less than 0.1%), and the specific surface area was 130 m^2^/g. The sterile raw material was suspended in M199 culture medium (Gibco, Grand Island, NY, USA) containing 10% fetal calf serum and then the suspension was sonicated at 180 W for 30 cycles, with 10 s ultrasonication and 5 s pause using an ultrasonic disrupter (JY92-IIN, Scientz, Ningbo, China). The suspensions were always prepared freshly before use. The MWCNT's microstructure in the solution was rigid and rod-like fiber with some occasional agglomerates, and the estimated diameters of MWCNTs ranged from 120 to 280 nm, and the estimated length ranged from 2 to 10 mm [10].

### 2.2. Cell Culture and Cytotoxicity Analysis

Human pleural mesothelial MeT-5A cells, purchased from the American Type Culture Collection (ATCC) (CRL-9444), were routinely subcultured in M199 culture medium containing 10% fetal bovine serum at 37°C and 5% CO_2_.

The short-term effects of MWCNT on cell viability were examined by lactate dehydrogenase (LDH) cytotoxicity assay kit (Beyotime, Shanghai, China) according to the manufacturer's instruction. Briefly, MeT-5A cells were firstly exposed to MWCNT at different concentrations (0, 1.25, 2.5, 5, 10, 20, and 40 *μ*g/cm^2^) for 24 h and then exposed to MWCNT at 10 *μ*g/cm^2^ for different periods (0, 24, 48, and 72 h). After treatment, the supernatant was removed to a 96-well plate and 60 *μ*L of the LDH test reagent was added to each well. Following a 30-minute incubation period, the absorbance at a wavelength of 490 nm was measured using SpectraMax M5 multimode microplate reader (Molecular Devices, Sunnyvale, USA). The cell viability was expressed as the percentage of the control which was without treatment.

For long-term treatment, MeT-5A cells were exposed to MWCNT at 10 *μ*g/cm^2^ throughout 3 months, the cell images were recorded if needed, and the cell number was counted by hemocytometer after various periods of treatments.

### 2.3. Immunofluorescence Microscopy

MeT-5A cells were subjected to MWCNT exposure at 10 *μ*g/cm^2^ throughout 3 months, and the formation of *γ*H2AX foci observed by Immunofluorescence microscopy was conducted essentially as described previously with slight modification [12]. Briefly, after treatments, 2 × 10^5^ cells were seeded into glass-bottom 6-well plates and allowed to grow to 70% confluence. Then, cells were fixed in 4% paraformaldehyde for 10 min, washed with PBS once, and permeabilized in 0.2% Triton X-100 for 5 min. After blocking for 1 h, samples were incubated with a mouse monoclonal anti-*γ*H2AX antibody (1 : 3000) (Upstate Technology, Lake Placid, NY) overnight at 4°C, followed by incubation with FITC-conjugated goat-anti-mouse secondary antibody (1 : 500) (Beijing Zhongshan Biotechnology Company, China) for 1 h. To stain the nuclei, Hoechst 33258 (Sigma, St. Louis, USA) was added to the cells and incubated for another 15 min in the dark. The glass-bottom 6-well plates were observed using a LSM710 Laser Scanning Confocal Microscope (Zeiss, Jena, Germany).

### 2.4. Wound-Healing Assay

To evaluate cell migration, a wound-healing assay was performed. MeT-5A cells plated in 35-mm dishes were exposed to MWCNT at 10 *μ*g/cm^2^ throughout 3 months. When cells grew to confluence, the cell monolayer was scratched to form a 100 *μ*m “wound” using sterile pipette tips and washed gently once with PBS. MeT-5A cells were then incubated with normal medium for another 24 h and 48 h. The wound was photographed at 0, 24 h, and 48 h using a DMI4000B microscope (Leica, Wentzler, Germany). The cell migration distance was measured by Image J software (National Institute of Mental Health, Bethesda, USA) at each time point. The ratio of the reduction of width at each time point to the initial width of scraped area (0 h) was expressed as percentage of migration at each time point.

### 2.5. Matrigel Invasion Assay

BD BioCoat Matrigel invasion chambers (24-well plate, BD, CA) were used to study the invasion activity of MeT-5A cells. After treatments by MWCNT at 10 *μ*g/cm^2^ for 0 h, 24 h, 48 h, 72 h, 1 month, and 3 months, 1 × 10^5^ cells were placed into the upper chamber of an insert coated with Matrigel in 200 *μ*l serum-free media and then incubated for another 48 h after adding 500 *μ*l media containing 10% FBS to the lower chamber. After incubation, the cells remaining on the upper membrane were removed with cotton swab, whereas the cells invaded through the membrane pores were stained with 0.1% crystal violet in methanol and visualized and counted using an inverted phase contrast microscope.

### 2.6. Western Blot Analysis

After treatments by MWCNT at 10 *μ*g/cm^2^ for 0 h, 8 h, 24 h, 48 h, 72 h, 1 month, and 3 months, control and MWCNT-treated groups of MeT-5A cells were lysed with Radio Immunoprecipitation Assay (RIPA) lysis buffer (Beyotime) supplemented with phenylmethanesulfonyl fluoride (PMSF) (Beyotime) and phosphatase inhibitor complex (Sangon Biotech, China) on ice for 40 minutes. The supernatant was collected after centrifugation at 13,000 rpm and 4°C for 15 minutes. The protein concentration in the supernatant was measured by the BCA protein assay (Bio-Rad, California, USA).

Equal amounts of proteins were loaded and separated by 10% SDS-PAGE and then transferred to PVDF membranes in transfer buffer (25 mM Tris, 200 mM glycine, 20% methanol v/v). The membranes were blocked with 5% BSA in TBST (Tris 20 mM, NaCl 137 mM, Tween-20 0.1%, pH 7.6) for 1 h at room temperature. After washing with TBST, the membranes were incubated in primary antibody at 4°C overnight followed by washing three times with TBST and incubation with the secondary antibody for 1 h at room temperature. Antibodies against Annexin 1 (BD, diluted 1 : 1000), Annexin 2 (BD, diluted 1 : 1000), Annexin 5 (Cell Signaling, diluted 1 : 1000), and Annexin 6 (BD, diluted 1 : 1000) and secondary antibodies HRP IgG (Multisciences, diluted 1 : 5000) were used. GAPDH (Santa Cruz, diluted 1 : 3000) was employed as an internal control. The protein bands were scanned using a FluorChem FC2 Imaging System (Alpha, San Antonio, USA).

### 2.7. siRNA Transfection

To investigate the role of Annexin 1 on cell migration, downregulation of Annexin 1 was conducted by siRNA interfering. Briefly, M-MeT-5A cells were transfected with 10 nM Annexin 1 siRNA (GenePharma) by using Lipofectamine 2000 reagent (Invitrogen) in Opti-MEM medium (Gibco) and incubated for 6 h at 37°C. Annexin 1 siRNA sequences are as follows: siRNA-1: GCAGCAUAUCUCCAGGAAATT; siRNA-2: GCUUUGCUUUCUCUUGCUATT; siRNA-3: GCCAUGAAAGGUGUUGGAATT. To exclude the possibility of off-target effects, cells were transfected with 10 nM Nontarget siRNA (GenePharma) as control. Inhibition of Annexin 1 expression by siRNA was confirmed by Western blotting as described above.

### 2.8. Statistics Analysis

Each experiment was conducted at least three times. Statistical analysis was performed using one-way ANOVA and Student's *t*-test. Numerical values are represented by mean ± SD. A statistical probability of *P* < 0.05 was considered significant.

## 3. Results

### 3.1. MWCNTs Induce Cytotoxicity in MeT-5A Cells at High Concentrations after Short-Term Treatments

**Figure 1:**
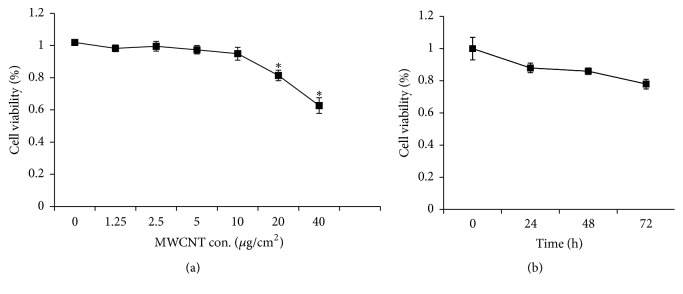
Cytotoxicity of MWCNT in MeT-5A cells. MeT-5A cells were seeded in 96-well plate (1 × 10^4^ cells/well) and subjected to various treatments. Cell viability was determined by the LDH assay and was expressed as the percentage of the control which was without treatment. Each experiment was repeated at least three times, and error bar stands for standard deviation (SD). (a) MeT-5A cells were treated with MWCNT at different concentrations (0, 1.25, 2.5, 5, 10, 20, and 40 *μ*g/cm^2^) for 24 h. (b) MeT-5A cells were treated with MWCNT for different times (0, 24, 48, and 72 h) at 10 *μ*g/cm^2^. ^*∗*^*P* < 0.05 versus control cells.

MeT-5A cells were treated by MWCNT at various concentrations (0, 1.25, 2.5, 5, 10, 20, and 40 *μ*g/cm^2^) for 24 h and the cytotoxicity was evaluated by LDH assay. As shown in Figure 1(a), the cell viability was suppressed significantly at concentrations ≥ 20 *μ*g/cm^2^ throughout the 24 h period compared with control cells. Then, MeT-5A cells were subject to MWCNT at 10 *μ*g/cm^2^ for 0, 24, 48, and 72 h, and although the cell proliferation rate showed the trend of decrease, no significant differences existed among the groups (Figure 1(b)). Accordingly, 10 *μ*g/cm^2^ was chosen for the subsequent experiments.

### 3.2. Long-Term MWCNT Exposure Alter Cell Morphology and Growth Pattern

**Figure 2:**
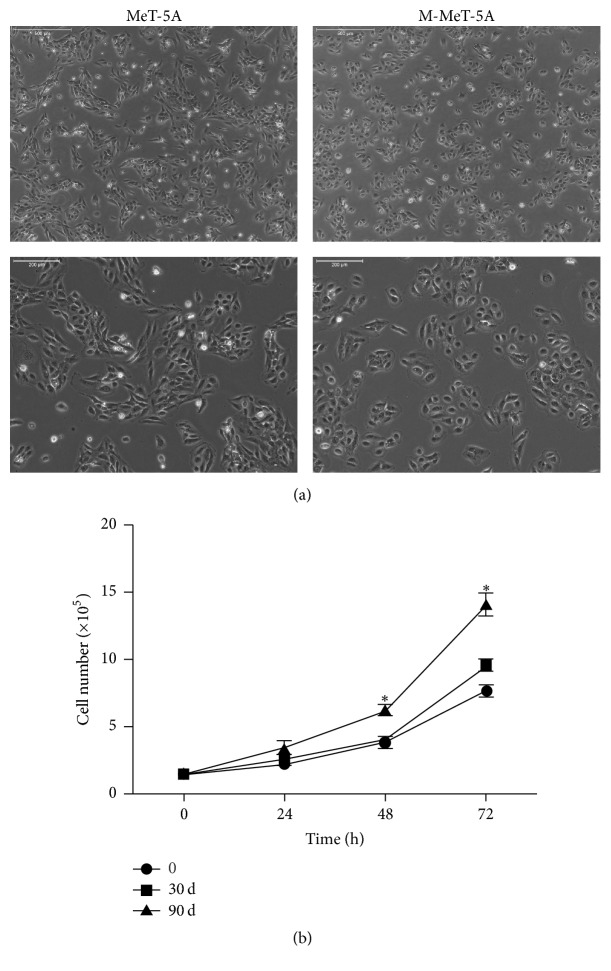
MWCNT induced morphological changes and increased growth of MeT-5A cells. Subconfluent cultures (1.5 × 10^5^ cells) of MeT-5A cells in 6-well plates were continuously exposed to 10 *μ*g/cm^2^ of MWCNT for 3 months. (a) Phase contrast micrographs of subconfluent monolayers of passage-matched control MeT-5A cells and MWCNT-treated cells at 12 weeks (M-MeT-5A). Scale bar = 500 *μ*m (upper images); scale bar = 200 *μ*m (lower images). (b) MeT-5A and MWCNT-treated cells (1 month and 3 months) were plated in 6-well plates at a density of 1.5 × 10^5^ cells in growth medium. After 24 h, 48 h, and 72 h, cells were digested and the cell number was counted by hemocytometer. Data are means ± SD (*n* = 3). ^*∗*^*P* < 0.05 versus passage-matched control cells.

Subconfluent cultures of MeT-5A cells were continuously exposed to MWCNT and passaged weekly at 10 *μ*g/cm^2^ for 3 months. Consequently, the long-term MWCNT-treated cells (designated as M-MeT-5A) exhibited a morphological change to be smaller and round in shape, whereas passage-matched control MeT-5A cells maintained the generally more expanded and elongated shape (Figure 2(a)). To determine whether chronic MWCNT exposure affects cell growth, the proliferative rate of M-MeT-5A and passage-control MeT-5A cells was evaluated by hemocytometer. M-MeT-5A cells showed a significant increase in cell proliferation rate above controls at 48 and 72 hours after seeding (Figure 2(b)).

### 3.3. MWCNTs Induce Genotoxicity in MeT-5A Cells

**Figure 3:**
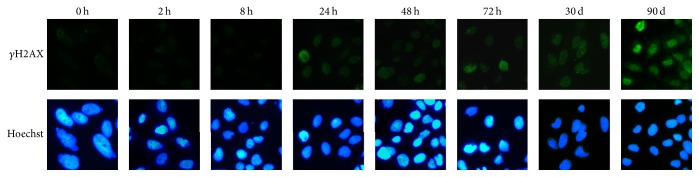
MWCNT causes DNA damage in MeT-5A cells. Following treatments of MWCNT at 10 *µ*g/cm^2^ for 0, 2 h, 8 h, 24 h, 48 h, 72 h, 1 month, and 3 months, cells were fixed and stained with anti-*γ*H2AX antibody and then subjected to immunofluorescent microscopy. Shown are representative images from three independent experiments (×100-fold). Blue, Hoechst 3358 stain for nuclei; Green, *γ*H2AX.

Although 10 *μ*g/cm^2^ of MWCNT did not cause significant cytotoxicity, we were wondering if any other types of toxicity, such as genotoxicity, could be elicited. To investigate DNA damage in MWCNT-treated MeT-5A cells, we applied the *γ*H2AX foci formation technique. The representative immunofluorescent images were shown in Figure 3. It was found that few *γ*H2AX foci formation could be observed in control cells and cells treated with MWCNT less than 24 h, but after treatment for more than 24 h to 3 months, the numbers of *γ*H2AX foci per cell and cell with *γ*H2AX foci both increased along with the increased exposure time.

### 3.4. MWCNTs Perturb Migration and Invasion of MeT-5A Cells

**Figure 4:**
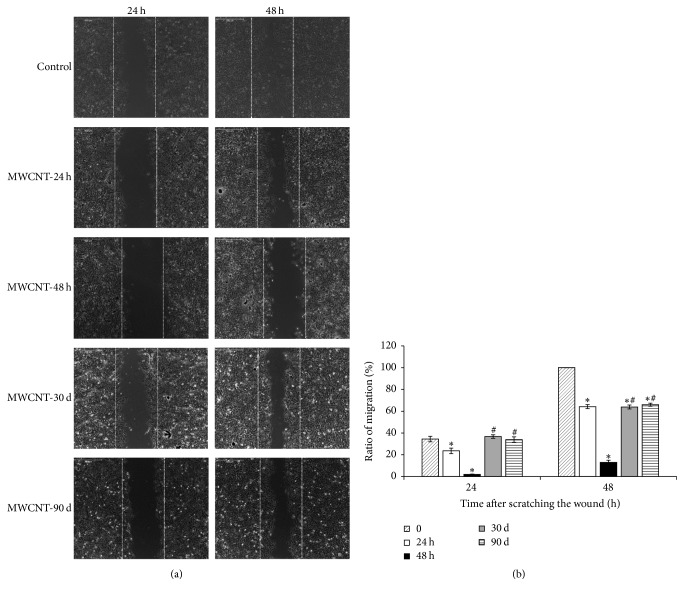
MWNCT affects cell migration. Control MeT-5A cells or MWCNT-treated cells (10 *µ*g/cm^2^ for 24 h, 48 h, 1 month, and 3 months) were grown to confluence, scratched, and then allowed to recover for 24 and 48 h. The dotted lines in all the images showed the locations of cells at time 0 h. (a) Representative images from three independent experiments. Scale bar = 500 *μ*m. (b) Quantitative data from (a) were presented as mean ± SD. ^*∗*^*P* < 0.05 versus control, ^#^*P* < 0.05 versus short-term MWCNT-treated cells (48 h).

Cell migration was analyzed using wound-healing assay. The results showed that MeT-5A cells displayed a significant reduction of cell migration after MWCNT exposure for 24 h and 48 h, as seen in Figure 4. Interestingly, the long-term MWCNT treatments attenuated the inhibition effects; MeT-5A cells exhibited faster migration to heal the scratched wound compared to those treated for short time periods, although it was still slower than normal cells. Further quantitation of wound-healing process showed that, at 24 and 48 h, the control MeT-5A cells had 34.4 ± 2.5% and 100% of migration rate, while the rates were 23.6 ± 2.5% and 64.2 ± 1.8% for 24 h MWCNT-treated cells and 2.0 ± 0.3% and 13 ± 1.9% for 48 h MWCNT-treated cells. For long-term exposure, the rates were 36.7 ± 1.6% and 63.9 ± 1.8% for 30-day MWCNT treatment and 33.9 ± 2.5% and 66.0 ± 1.4% for 90-day MWCNT treatment, respectively.

**Figure 5:**
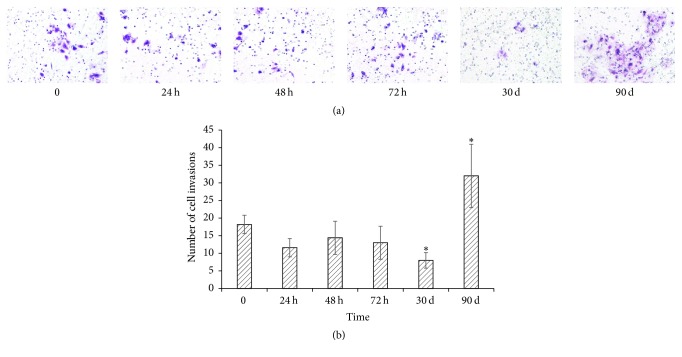
MWNCT perturbs cell invasion of MeT-5A cells. MeT-5A cells were exposed to MWCNT at 10 *µ*g/cm^2^ for 24 h, 48 h, 72 h, 1 month, and 3 months. After treatments, cells were incubated in the BD BioCoat Matrigel invasion chambers to study the invasion activity. (a) Representative images from three independent experiments (×200-fold). (b) Quantitative data from (a) were presented as mean ± SD. ^*∗*^*P* < 0.05 versus control.

Invasion growth is another key feature of malignant transformation. Cell invasion was analyzed by Matrigel-coated membranes approach. As shown in Figure 5, MWCNT caused a decreased trend of cell invasion within 72 h exposure, and at 30 days, it was decreased to 44% compared to that of control MeT-5A cells (*P* < 0.05). But after 3 months of MWCNT exposure, the invasion ability was reversed, and it was increased to almost 2-fold that of the control cells (*P* < 0.05).

### 3.5. MWCNTs Induce Changes in Annexin Family Proteins Expression

**Figure 6:**
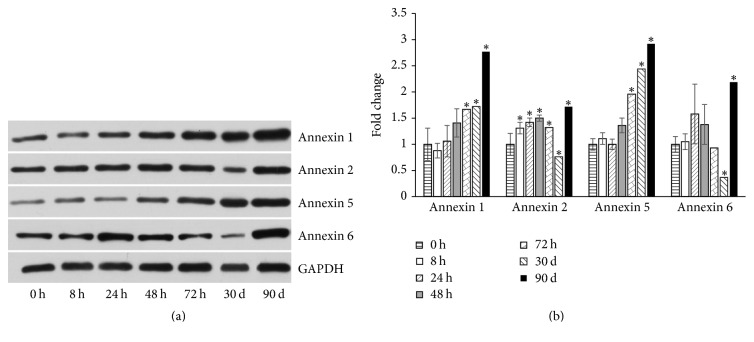
MWCNT significantly changes the Annexin proteins expression. (a) Western blot results for Annexin 1/2/5/6 expression in MeT-5A cells treated with 10 *µ*g/cm^2^ for 8 h, 24 h, 48 h, 72 h, 1 month, and 3 months. (b) Densitometry analysis of (a). GAPDH was used as a loading control. ^*∗*^*P* < 0.05.

It has been reported that some Annexin proteins are involved in cell proliferation, cell migration, tumor cell metastasis, and so on. The expression patterns of Annexin 1, Annexin 2, Annexin 5, and Annexin 6 were detected in MeT-5A cells after MWCNT treatments in our study.  Figure 6 showed the representative images of these four proteins with expression changes. Annexin 1 and Annexin 5 basically exhibited a dose-dependent increase in protein expression levels. On the other hand, the changes for Annexin 2 and Annexin 6 were relatively complex, both of which had a sharp decrease at 30 d and then increased at 90 d.

### 3.6. Knockdown of Annexin 1 Decreases Cell Migration in M-MeT-5A Cells

**Figure 7:**
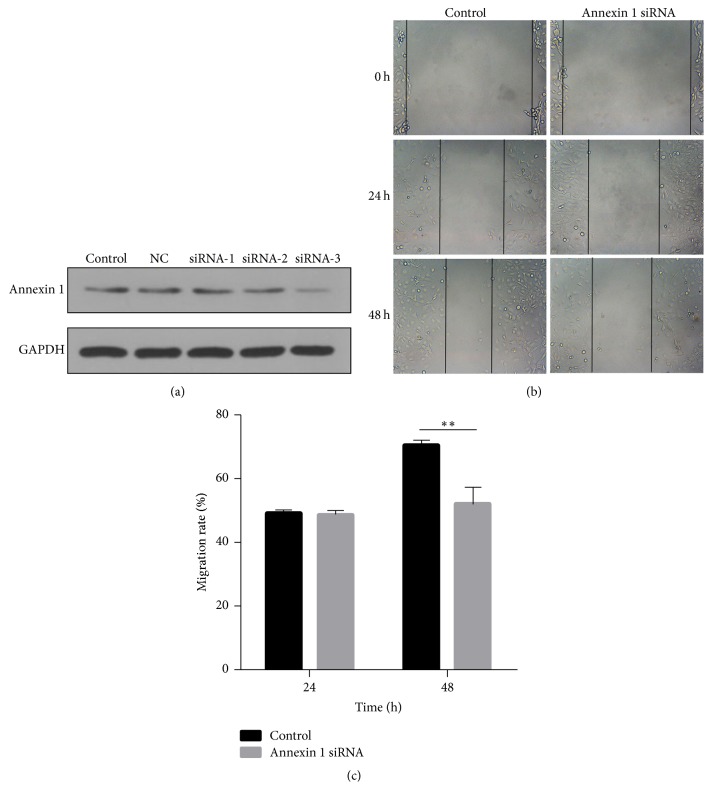
Knockdown of Annexin 1 decreases cell migration in M-MeT-5A cells. (a) Annexin 1 expression is downregulated significantly by siRNA-3. Three Annexin 1 siRNA sequences were applied, and their effects on Annexin 1 expression were examined by Western blotting. (b) Si-Annexin 1 or si-Control transfected M-MeT-5A cells were grown to confluence, scratched, and allowed to recover for 48 h. Shown are representative images of cell migration from three independent experiments (×100-fold). (c) Quantitative data of (b) were presented as mean ± SD. ^*∗∗*^*P* < 0.01 versus control.

Annexin 1 was significantly downregulated by siRNA-3 sequence, compared to the other two ones, as shown in Figure 7(a). The effects of Annexin 1 on cell migration were measured by cell scratch analysis. Consequently, cell migration was significantly suppressed by Annexin 1 downregulation. As shown in Figures 7(b) and 7(c), at 24 h the calculated migration rates were almost the same for si-Annexin 1 cells and si-Control cells. However, at 48 h, the calculated migration rate was about 50% for si-Annexin 1 cells and 70% for si-Control cells.

## 4. Discussion

MWCNT has been linked to asbestos in terms of morphology and toxicity, which could lead to lung cancer or even mesothelioma [4, 13], but contradictory findings coexist. Possible explanations include the different cell systems, varied types of commercial MWCNTs, different detection time points, and different MWCNT concentrations applied in these studies. For example, MeT-5A cells were more sensitive to the DNA-damaging effect than BEAS-2B cells, despite the fact that more CNT fibers or clusters were seen in BEAS-2B than those in MeT-5A cells [14]. Low doses of ND-MWCNT (1.2 *μ*g/mL) or MWCNT-7 (0.12 *μ*g/mL) increased cellular proliferation, while the highest dose of 120 *μ*g/mL of either material decreased the proliferation, and repeated exposure is more damaging than a single exposure [15]. Short tube length MWCNT has more capacity to induce genotoxicity because of its persistent presence in cells [16]. Furthermore, time length of MWCNT exposure is also an important matter; for instance, a 48 h exposure of NM-402 MWCNT did not cause cytotoxic effects in A549 cells, but after 8 d exposure, cytotoxic effects were clearly found in A549 cells [7], which is similar to our findings in this study. Human pleural mesothelial cells (MeT-5A) are the primary cellular target of mesothelioma; therefore we used MeT-5A cells as the model system to investigate the effects of MWCNT exposure. As a result we have found the different cellular responses to MWCNT after short-term or long-term treatments. For instance, MWCNT had no effect on cell proliferation at 10 *μ*g/cm^2^ during a 72 h period, but after 3 months of sustained exposure, the cell proliferation rate was significantly increased, for which the abnormal cell growth is considered to be one of the hallmarks of carcinogenesis [17]. Moreover, after long-term treatments, MeT-5A cell morphology was also changed to be smaller and round in shape, which is consistent with the results for human lung epithelial BEAS-2B cells after exposure to single-walled carbon nanotube [18]. It has been reported that MWCNT-induced formation of polyploidization and aneuploidization may be attributed to the cell morphology changes [13]. In our earlier study, MWCNT caused increased expression of actin as well as actin filament remodeling in A549 cells, which may be another reason for the morphology change [12]. Furthermore, Annexin 1 and its family member Annexin 2 could act as candidate regulators of oncogene-induced cell morphology switch [19].

DNA alteration is another characteristic of carcinogenesis. The effect of MWCNT on *γ*H2AX foci formation was examined in our study. As expected, the numbers of *γ*H2AX foci per cell and cell with *γ*H2AX foci increased along with the increased exposure time. Similarly, exposure of human umbilical vein endothelial cell (HUVEC) to MWCNT also increased the *γ*H2AX-positive cells in a dose-dependent manner (0.5–20 mg/mL) [20]. The genotoxicity of MWCNT has been shown to result predominantly from oxidative stress induced by excessive inflammatory responses to CNT fibers [21].

Cell migration is a crucial step in many physiological or pathological processes such as wound-healing, cancer, and inflammation [22]. It is reported that long MWCNT (20 *μ*m) reduced the migratory capacity in primary human alveolar macrophages (AMs) along with increased expression of MARCO [23]. In contrast, MWCNT promoted cell migration in RAW263.7 macrophage cell lines and human microvascular endothelial cells (HMVEC) [24, 25]. Our previous study also showed an increasing migration capacity of A549 cells after MWCNT exposure for 24 h at 30 *μ*g/mL [12], consistent with the finding in Pacurari et al.'s study [26]. In this study, we investigated the effect of short-term and long-term exposure of MWCNT on the migration behaviors to MeT-5A cells. Interestingly, short-term exposure reduced the migration ability, while the longer exposure (3-month) tended to reverse the effects, even though to a less degree of cell movement compared to control. The biphasic development with such drastically distinct wound-healing activity suggests a junction of acute-to-chronic transition, which may reflect the chronic and accumulated toxicity of MWCNT under sustained exposure to cells. Cell invasion refers to the three-dimensional migration of cells as they penetrate an extracellular matrix (ECM) and is a process typically associated with cancer cell metastasis [27]. It has been reported that MWCNT-exposed MeT-5A cells demonstrated a significant increase in cell invasion (5.4- to 6.3-fold) and migration (2.5- to 2.7-fold) as compared to Survanta-treated control [28]. Long-term (6-month) exposure of the human lung small airway epithelial cells (SAECs) to CNT also caused a neoplastic-like transformation phenotype as demonstrated by increased cell proliferation, anchorage-independent growth, invasion, and angiogenesis [29]. Likewise, 3 months of MWCNT exposure to MeT-5A cells also resulted in increased invasion capacity in our study. Still, there were not many cells that penetrated to the bottom cell box, indicating a relatively weak invasion capacity, which may be one of the explanations that mesothelioma had a low rate of distant metastasis [30].

As noted above, MWCNT exposure affects the migration and invasion ability of MeT-5A cells. To elucidate the underlying mechanism, we investigated the possible involvement of Annexin proteins. Annexin family members have been shown to be differentially expressed in epithelial malignancies and are thought to regulate critical cellular processes involved in malignant transformation and/or progression of neoplastic diseases [31, 32]. Annexin 1 is a multifunctional phospholipid-binding protein associated with the development of metastasis in some invasive epithelial malignancies. Overexpression of Annexin 1 increased cell migration and invasion in esophageal squamous cell carcinoma (ESCC) and gastric cancer cell [33, 34]. Knockdown of Annexin 1 expression resulted in a significant reduction in invasion in colorectal adenocarcinoma epithelial cells SKCO-15 [35]. Similarly, in our study, downregulated Annexin 1 decreased cell migration in MeT-5A cells. Other members of the Annexin family have also been implicated in cell migration. For example, Annexin 2^+^ adamantinomatous craniopharyngioma (AdaCP) cells exhibited enhanced proliferation and migration ability compared with Annexin 2^−^ AdaCP cells in vitro [36]. Upregulated Annexin 2 promoted the proliferation, migration, and invasion of Caco-2 cells in vitro, in association with STAT3 [37]. Annexin 5 knockdown also resulted in significantly reduced proliferation, migration, invasion, and in situ lymph node adhesion potentials of hepatocarcinoma Hca-F cells in proportion to its knockdown extent [38]. The specific role of Annexin 6 in cell migration depends on the type of cancer and the level of malignancy [39]. Loss of Annexin 6 suppresses the invasiveness and motility of breast cancer (BC) and BC cells, while enhancing the anchorage-independent cell growth of BC cells [40]. In our study, the changes of Annexin 2 and 6 were complex, and the reasons for that are not clear. On the other hand, both Annexin 1 and 5 were increased after either short- or long-term MWCNT exposure; however, cell migration ability was inhibited, which seemed to contradict the function of Annexin proteins. One possible explanation is that different type of cells could have a different response to the same material, for example, A549 cells in our previous study [12] and MeT-5A in this study. Also, the MWCNTs used in our previous study were synthesized by Lawrence Berkeley National Laboratory, while the MWCNTs used in this study were purchased from Aldrich. Actually the many different kinds of CNTs (single-walled, double-walled, and multiwalled) could all have different effects on cells. Even the shape of the CNTs, such as the rigid and rod-like or tangled MWCNT, may influent their biological outcome as these two types tend to have very different effects both in vitro and in vivo. For example, only the so-called Mitsui-7 MWCNTs, rigid and rod-like, have induced mesothelioma-like pathogenicity in rodents. This is the same material that was classified into category 2B by IARC. Still, there must be some other factors contributing to such discrepancy, which needs to be further studied in detail.

## 5. Conclusion

The cellular responses of MeT-5A to short-term and long-term MWCNT treatments are different, and after long-term exposure, MeT-5A cells exhibit certain characteristics related to carcinogenic potential. Specifically, cell motility, including cell migration and cell invasion, is perturbed by MWCNT, in which Annexin proteins might be involved.
